# GABAergic synaptic response and its opioidergic modulation in periaqueductal gray neurons of rats with neuropathic pain

**DOI:** 10.1186/1471-2202-12-41

**Published:** 2011-05-12

**Authors:** Eu-Teum Hahm, Younghoon Kim, Jong-Ju Lee, Young-Wuk Cho

**Affiliations:** 1 Department of Physiology, Biomedical Science Institute, Kyung Hee University School of Medicine, Seoul 130-701, South Korea; 2 Department of Physiology and Biophysics, University of Colorado Denver, Anschutz Medical Campus, Aurora, CO 80045, USA; 3 Department of Dental Pharmacology, School of Dentistry, Kyungpook National University, Daegu 700-412, South Korea

**Keywords:** Neuropathic pain, Endogenous pain control system, Opioid analgesia, GABAergic synaptic transmission, Periaqueductal gray

## Abstract

**Background:**

Neuropathic pain is a chronic and intractable symptom associated with nerve injury. The periaqueductal gray (PAG) is important in the endogenous pain control system and is the main site of the opioidergic analgesia. To investigate whether neuropathic pain affects the endogenous pain control system, we examined the effect of neuropathic pain induced by sacral nerve transection on presynaptic GABA release, the kinetics of postsynaptic GABA-activated Cl^- ^currents, and the modulatory effect of μ-opioid receptor (MOR) activation in mechanically isolated PAG neurons with functioning synaptic boutons.

**Results:**

In normal rats, MOR activation inhibited the frequency of GABAergic miniature inhibitory postsynaptic currents (mIPSCs) to 81.3% of the control without any alteration in their amplitude. In neuropathic rats, the inhibition of mIPSC frequency by MOR activation was 82.4%. The frequency of GABAergic mIPSCs in neuropathic rats was 151.8% of normal rats without any difference in the mIPSC amplitude. Analysis of mIPSC kinetics showed that the fast decay time constant and synaptic charge transfer of mIPSCs in neuropathic rats were 76.0% and 73.2% of normal rats, respectively.

**Conclusions:**

These results indicate that although the inhibitory effect of MOR activation on presynaptic GABA release is similar in both neuropathic and normal rats, neuropathic pain may inhibit endogenous analgesia in the PAG through an increase in presynaptic GABA release.

## Background

Chronic pain can be classified into three categories: nociceptive pain caused by tissue damage, neuropathic pain caused by nerve injury, and mixed pain [1]. Patients with neuropathic pain usually experience abnormal sensations, including allodynia, which is defined as pain in response to non-nociceptive stimuli and hyperalgesia, which is defined as increased pain sensitivity to nociceptive stimuli [2]. Although opioid receptor agonists are the most widely used therapeutic agents for neuropathic pain, the effectiveness of opioid analgesia is controversial. Several studies have shown that neuropathic pain can be effectively attenuated by morphine and other μ-opioid receptor (MOR) agonists [3-10] as well as delta-opioid agonists [11-14]. By contrast, some studies have indicated that opioid peptides and morphine do not possess potent analgesic efficacy against neuropathic pain in humans [15] and that this ineffectiveness of morphine can be attributed to a down-regulation of central μ-opioid transmission [16] and a reduced number of presynaptic opioid receptors due to the degeneration of primary afferent neurons [17,18].

The midbrain periaqueductal gray (PAG) is believed to be an important component in the endogenous pain control system [19]. Several studies have shown that administration of morphine or opioid peptides, either systemically or directly into the PAG, produces antinociception, which is thought to be associated with inhibition of neuronal activity in the PAG [20,21]. The inhibitory interneurons in the PAG are thought to contain GABA as an inhibitory neurotransmitter and inhibit tonically the output neurons [19,22-25]. Opioid agonists have been shown to inhibit GABAergic inhibitory synaptic input to PAG neurons in rat slice preparations [24,25]. In previous studies, we have shown that MOR activation inhibits presynaptic GABA release in acutely isolated PAG neurons from normal young rats [26].

Although many studies have investigated the analgesic effects of opioid agonists on neuropathic pain, it is not clear whether opioidergic modulation of the endogenous pain control system in the PAG is altered by neuropathic pain. Therefore, in the present study, we isolated PAG neurons with intact synaptic terminals from rats with neuropathic pain to examine the effects of neuropathic pain on presynaptic GABA release, the kinetics of postsynaptic GABA-activated Cl^- ^currents, and the opioid-induced inhibition of GABAergic synaptic action.

## Results

### GABAergic mIPSCs in PAG neurons isolated from neuropathic rats

There were no differences in morphological characteristics between normal and neuropathic rats. Mechanically dissociated PAG neurons retained short portions of their proximal dendrites and usually presented an ovoid soma (approximately 10-20 μm in diameter), although some neurons presented a triangular soma.

**Figure 1:**
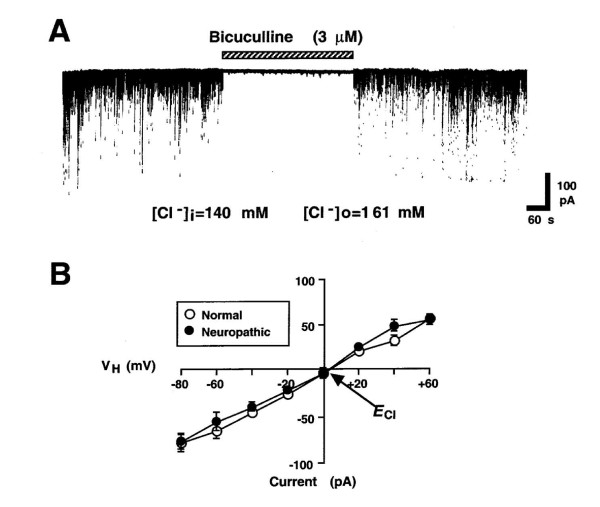
**GABAergic spontaneous mIPSCs in PAG neurons isolated from normal and neuropathic rats**. (A) In the presence of 300 nM TTX, 3 μM CNQX and 10 μM AP5, the application of 3 μM bicuculline completely and reversibly blocked mIPSCs in PAG neurons isolated from normal rats. The holding voltage (V_H_) was -60 mV. (B) I-V curve obtained from normal (open circle) and neuropathic rats (closed circle) for the mean amplitude of mIPSCs recorded at various V_H_. Each point is the mean of four neurons. *E*_Cl _is the reversal potential of mIPSCs.

We recorded and measured the mean amplitude and frequency of mIPSCs 20 min after the rupture of the patch membrane because it took 10-20 min for synaptic currents to stabilize (Figure 1). In most neurons, recordings of these mIPSCs were stable for approximately 60 min, which indicated that the presynaptic nerve terminals attached to the dissociated PAG neurons were functional and that their spontaneous activity was stable for at least 60 min. In normal rats, the application of 3 μM bicuculline, a GABA_A _receptor antagonist, completely and reversibly blocked mIPSCs (Figure 1A). Reversal potentials and the slopes of the current-voltage (I-V) curves were almost identical in both groups. The reversal potential (-5.2 mV for normal rats, -4.9 mV for neuropathic rats) of these mIPSCs, as estimated from the current-voltage (I-V) relationship, was very similar to the theoretical Cl^- ^Nernst equilibrium potential (E_Cl_) of -3.5 mV, calculated using extracellular and intracellular Cl^- ^concentrations of 161 and 140 mM, respectively (Figure 1B). The conductance of mIPSCs was 0.924 ± 0.13 μS for normal rats and 0.859 ± 0.11 μS for neuropathic rats (*n *= 9, *P *= 0.715). These results indicate that spontaneous mIPSCs in normal rats are mediated by GABA_A _receptors.

### Effect of MOR activation on GABAergic mIPSCs in neuropathic rats

**Figure 2:**
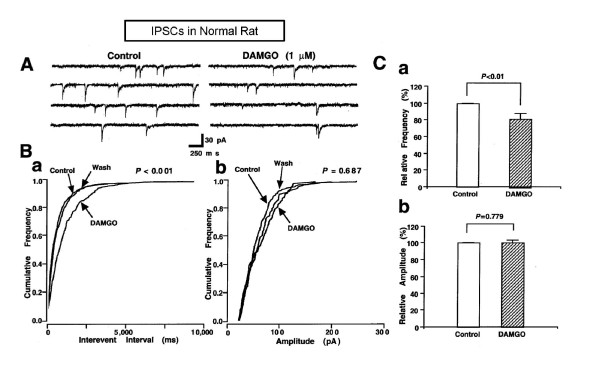
**Inhibitory effect of μ-opioid receptor activation on GABAergic mIPSCs in normal rats**. (A) Representative recording trace of mIPSCs before and during the application of DAMGO (1 μM). (B) Histograms showing cumulative frequency (a) and amplitude distribution (b) of mIPSCs inhibited by DAMGO. (C) Each column is the mean of 49 neurons. All frequencies (a) and amplitudes (b) are normalized to those of control mIPSCs.

In a previous report, we indicated that MOR activation reduces GABAergic mIPSC in PAG neurons of young rats [26]. In normal rats, application of 1 μM DAMGO, a specific MOR agonist, also reduced the frequency of GABAergic synaptic events in the majority of PAG neurons tested (61%, 30 of 49 neurons). In the remaining 19 neurons, DAMGO did not significantly alter the frequency or amplitude of mIPSCs. The DAMGO-induced decrease in mIPSC frequency was reversible (Figure 2) and could be blocked by pretreatment with 3 μM naloxone, a non-specific opioid receptor antagonist (100.5 ± 17.0% of the control, *n *= 3, *P *= 0.981, data not shown). DAMGO decreased the mIPSC frequency to 81.3 ± 6.4% of the control (Figure 2Ba, Ca; *n *= 49, *P *< 0.01), but did not alter their amplitude distribution (Figure 2Bb, Cb; 99.4 ± 2.0% of controls, *n *= 49, *P *= 0.779). These data indicate that DAMGO inhibits presynaptic GABA release via MOR activation at presynaptic terminals in PAG neurons of normal rats.

**Figure 3:**
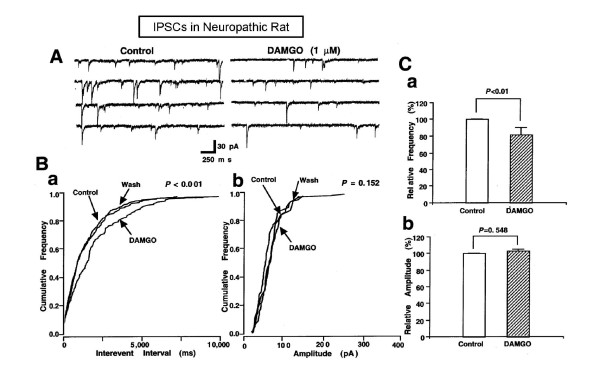
**Inhibitory effect of μ-opioid receptor activation on GABAergic mIPSCs in neuropathic rats**. (A) Representative recording trace of mIPSCs before and during the application of DAMGO (1 μM). (B) Histograms showing the cumulative frequency (a) and amplitude distribution (b) of mIPSC inhibited by DAMGO. (C) Each column is the mean of 35 neurons. All frequencies (a) and amplitudes (b) were normalized to those of control mIPSCs.

Although statistically not significant, the number of neurons showing the inhibitory effect of DAMGO on the frequency of mIPSCs was lower in neuropathic rats (54%, 19 of 35 neurons) than in normal rats (61%, 30 of 49 neurons; Fisher's Exact test, *P *= 0.654). The shape of these DAMGO-responsive neurons was similar in both the neuropathic and normal rats. The DAMGO-induced inhibition of mIPSC frequency was also reversible and could be blocked by pretreatment with 3 μM naloxone (data not shown). In neuropathic rats, DAMGO decreased the frequency of mIPSC to 82.4 ± 4.6% of controls (Figure 3Ba, Ca; *n *= 35, *P *< 0.05), but did not change the distribution of their amplitudes (Figure 3Bb, Cb; 102.1 ± 3.5% of controls, *n *= 35, *P *= 0.548). The inhibitory effect of DAMGO on mIPSC frequency was very similar in the neuropathic and normal rats. These results indicate that neuropathic pain does not alter the inhibitory effect of MOR activation on presynaptic GABA release in PAG neurons.

### Effect of neuropathic pain on presynaptic GABA release

**Figure 4:**
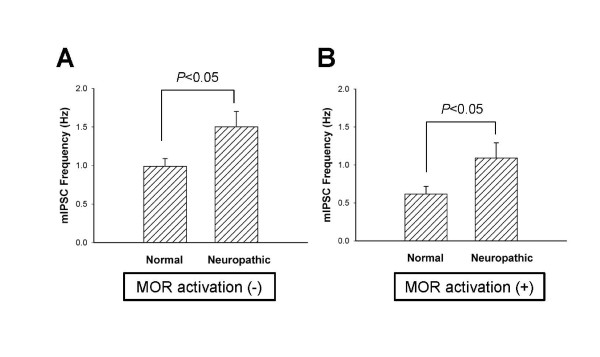
**Effect of neuropathic pain on presynaptic GABA release**. (A) Histograms comparing the frequency of mIPSCs between normal and neuropathic rats in the absence of 1 μM DAMGO. Each column is the mean of 49 neurons. (B) Histograms comparing the frequency of mIPSCs between normal and neuropathic rats in the presence of DAMGO. Each column is the mean of 35 neurons.

To investigate whether neuropathic pain alters presynaptic GABA release in PAG neurons as a mechanism of hyperalgesia and/or allodynia, we compared the frequencies of presynaptic GABA release in neuropathic and normal rats. As shown in Figure 4, the frequency of mIPSCs in neuropathic rats (1.503 ± 0.2 Hz, 151.8% of the normal rats, *n *= 35) was higher than that in normal rats (0.990 ± 0.1 Hz, *n *= 49; *P *< 0.05). In the presence of the inhibitory effect of DAMGO, neuropathic pain increased the frequency of mIPSCs from 0.616 ± 0.1 Hz in normal rats (*n *= 49) to 1.091 ± 0.2 Hz in neuropathic rats (*n *= 35, 177.1% of the normal rats; *P *< 0.05). These results indicate that neuropathic pain may increase presynaptic GABA release.

### Effect of neuropathic pain on the kinetics of postsynaptic GABA-activated Cl^- ^channels

**Figure 5:**
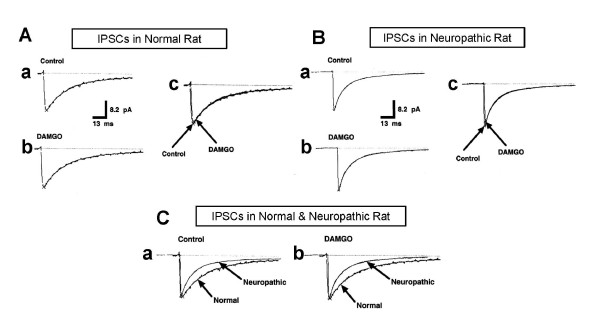
**Effect of DAMGO on GABAergic mIPSC kinetics in normal and neuropathic rats**. (A) Effect of DAMGO on GABAergic mIPSC kinetics in normal rats. (a) The average current of the control (Control), DAMGO-treated (b), and superimposed (c) mIPSCs in normal rats. There is no significant difference between control and DAMGO-treated currents. (B) Effect of DAMGO on GABAergic mIPSC kinetics in neuropathic rats. The control (a) and DAMGO-treated (b) currents of the averaged mIPSCs of neuropathic rats, and the superimposed current of the mIPSCs (c). There is no significant difference between control and DAMGO-treated currents. (C) Comparison of the kinetics of mIPSC modulated by neuropathic pain in the presence and absence of DAMGO. The superimposed currents of control (a) and DAMGO-treated (b) mIPSCs in normal and neuropathic rats.

To elucidate the effect of neuropathic pain on the kinetics of postsynaptic GABA-activated Cl^- ^channels, the kinetics of GABAergic mIPSCs were analyzed by fitting the currents with two exponential functions and were then described with time constants (Table 1 and Figure 5). The 10-90% rise time constant of normal rats (1.07 ± 0.1 ms) was not different from that of neuropathic rats (0.91 ± 0.1 ms, *P *= 0.066, Table 1). The weighted mean decay time constant of neuropathic rats (17.44 ± 0.9 ms) was significantly faster than that of normal rats (21.03 ± 1.2 ms, *P *< 0.05). In particular, while there was no difference between the slow decay time constants (τ_slow_) in both groups (39.89 ± 2.2 ms for normal rats, 36.64 ± 2.0 ms for neuropathic rats, Table 1), the fast decay time constant (τ_fast_) of neuropathic rats (8.92 ± 0.4 ms) was faster than that of normal rats (11.74 ± 0.8 ms, *P *< 0.05, Table 1). Due to the reduced fast decay time constant, the half-width time of mIPSCs in neuropathic rats could be shortened. Indeed, the half-width time constant in neuropathic rats (10.29 ± 0.5 ms) was shorter than that in normal rats (13.29 ± 0.8 ms, *P *< 0.05, Table 1). In addition, we calculated the area under the mIPSCs (synaptic charge transfer), which indicates the integration of the single channel open time and the duration of the open time of Cl^- ^channels [27,28]. The synaptic charge transfer was significantly reduced in neuropathic rats (753.6 ± 42.5 pAms for normal rats, 551.4 ± 35.4 pAms for neuropathic rats, *P *< 0.05, Table 1, Figure 5). By contrast, MOR activation with DAMGO showed no effect on mIPSC kinetics in neuropathic rats (Table 1, Figure 5). These results indicate that neuropathic pain may promote faster inactivation kinetics of postsynaptic GABA-activated Cl^- ^channels.

**Table 1:** The kinetics of mIPSC in normal adult and neuropathic rats

			Normal adult	Neuropathic
			(Mean ± S.E.)	(Mean ± S.E.)
**Rise 10-90% (ms)**		**Control**	1.07 ± 0.1	0.91 ± 0.1
		**DAMGO**	1.10 ± 0.1	0.94 ± 0.0
**Decay 90-37% (ms)**	**τ**_**fast**_	**Control**	11.74 ± 0.8	8.92 ± 0.4†
		**DAMGO**	13.32 ± 1.3	9.69 ± 0.6†
	**τ**_**slow**_	**Control**	39.89 ± 2.2	36.64 ± 2.0
		**DAMGO**	46.50 ± 3.9	36.99 ± 2.5
**Weighted mean decay time (ms)**		**Control**	21.03 ± 1.2	17.44 ± 0.9†
		**DAMGO**	23.55 ± 1.8	18.65 ± 1.0†
**Half-width (ms)**		**Control**	13.29 ± 0.8	10.29 ± 0.5†
		**DAMGO**	14.66 ± 1.1	11.52 ± 0.6†
**Area under currents (pAms)**		**Control**	753.6 ± 42.5	551.4 ± 35.4†
		**DAMGO**	897.0 ± 81.4	567.0 ± 39.1†

Values are mean ± S.E. † *P *< 0.05 for neuropathic rat compared to that of normal adult rats (Normal adult rat, *n *= 49 and neuropathic rat, *n *= 35).

## Discussion

The present study was performed to examine whether neuropathic pain alters presynaptic GABA release and postsynaptic GABA-activated Cl^- ^currents and whether opioidergic modulation of the GABAergic inhibitory synaptic response might be affected by neuropathic pain. Our results show that neuropathic pain increases the frequency of presynaptic GABA release and decreases both the fast decay time constant and the synaptic charge transfer of postsynaptic GABA-activated Cl^- ^currents, regardless of whether MOR agonists are present. In addition, neuropathic pain did not alter the inhibitory effect of MOR activation on GABAergic mIPSCs.

Neuropathic pain associated with peripheral neuropathy can manifest as severe and intractable pain. However, the mechanism of this severe and intractable pain remains unclear. The PAG is an important component of the endogenous pain control system and is the main site of the powerful analgesic effects by morphine or opioid peptides [19]. In a previous report, we suggested that MOR-induced inhibition of GABAergic inhibitory synaptic influence in the PAG is the main mechanism of the opioidergic endogenous pain control system [26]. If GABAergic synaptic inhibition of PAG neurons is potentiated by neuropathic pain, this may represent a potential mechanism of neuropathic pain. Although the exact mechanisms are not clear, there have been reports supporting this hypothesis. Activation of the descending pain control system was shown to be important in the maintenance of neuropathic pain [29]. The PAG-mediated inhibition of nociception may be activated by persistent nociceptive input, possibly reflecting the long-term changes in the nociceptive circuitry that occur in neuropathic pain states [30]. In this study, neuropathic rats showed an increase in the frequency of presynaptic GABA release in PAG neurons (Figure 4). This finding indicates that endogenous pain control mechanisms in the PAG may be inhibited in animals suffering from neuropathic pain. Thus, this study suggests that neuropathic pain inhibits the efficiency of the endogenous pain control system in the PAG, thereby inducing severe and intractable pain.

Although neuropathic pain does not alter the amplitude of postsynaptic GABAergic response, the kinetics of mIPSC was slightly inhibited in neuropathic rats (Figure 5, Table 1). Neuropathic rats showed a reduction in the fast decay time with a reduced half-width time and synaptic charge transfer of mIPSCs. These findings might indicate that GABAergic inhibitory input to the PAG neurons can be decreased in neuropathic rats, which means that endogenous pain control mechanisms in the PAG may be activated in neuropathic rats. However, the decrease in the fast decay time and synaptic charge transfer of mIPSCs (76.0% and 73.2% of the normal rats, respectively) was significantly less than the increase in presynaptic release of GABA (151.8% of the normal rats, Figure 4). Thus, the changes in mIPSC kinetics in neuropathic rats may not show a significant influence to the inhibitory effect of the decreased presynaptic GABA release in neuropathic rats on endogenous pain control mechanisms in the PAG.

Morphine and opioid peptides exert their powerful analgesic effects through the endogenous pain control system, especially in the PAG [19]. The efficiency of opioid receptor agonists, especially the MOR agonist morphine, has been reported in recent studies of central and peripheral neuropathic pain disorders [31-34]. However, the development of long-term side effects, such as immunological problems, physical dependency, and misuse or abuse, is a limitation to the use of opioid analgesics in patients with neuropathic pain [2]. Furthermore, and the effectiveness of opioid agonists on neuropathic allodynia and hyperalgesia remains controversial. Several studies have supported the effectiveness of opioid receptor agonists on neuropathic pain [3-14]. However, some studies have raised questions about the efficiency of opioid analgesics on neuropathic pain in humans [15] and animals [16-18,35]. Other studies have indicated that the PAG is important in opioidergic analgesia of neuropathic pain. Neuropathic pain that is induced by peripheral nerve injury has been effectively alleviated by electrical stimulation of the PAG [36], microinjection of opioid agonists into the PAG [37], and supraspinal administration of morphine into the PAG [9]. Although the analgesic mechanisms have not been clearly elucidated, these studies suggest that the endogenous pain control system, including the PAG, is very important in the control of neuropathic pain syndrome and that opioid receptors are involved in this system. In the present study, MOR agonists inhibited GABAergic inhibitory synaptic activity in the PAG of neuropathic rats, and this inhibitory effect of MOR activation was not significantly different between neuropathic and normal rats (Figure 2, 3, and 4). Thus, the results of this study suggest that MOR agonists can effectively exert an analgesic effect on neuropathic pain through the modulation of the endogenous pain control system in the PAG and that the analgesic effectiveness of opioid peptides in neuropathic animals is similar to that in normal animals. However, because neuropathic pain may inhibit the endogenous pain control mechanism in the PAG in the resting state (as described above), it is possible that the analgesic action of exogenous opioid agonists is less effectively in a neuropathic pain state.

While the majority of proximal dendrites are still attached to the neurons, the mechanical dissociation can alter the majority of distal dendrites. Although the remaining dendritic as well as somatic synapses are well elucidated to be still functioning [38], it cannot be ruled out that the dendritic synapses may be modulated in a different manner shown in the present study.

## Conclusions

The results of this study suggest that neuropathic pain inhibits the endogenous pain control system through an increase in presynaptic GABA release in the PAG, which then induces severe and intractable pain. Thus, although the effect of MOR activation on presynaptic GABA release in neuropathic rats is similar to that in normal animals, exogenous opioid agonists may exert their analgesic actions less effectively in neuropathic rats.

## Methods

### Animals and surgical procedures

All experimental protocols were approved by the Institutional Animal Care and Use Committee of the Kyung Hee University and all efforts were made to minimize animal suffering and the number of animals utilized. Male Sprague-Dawley rats (8-12 weeks old) were subjected to a neuropathic pain model, as described in detail previously [39,40]. In brief, the tail response to a mechanical stimulus was first tested in all animals prior to surgery. The rats were restrained in a transparent plastic tube (5 cm in diameter × 20 cm in length), and their tails were laid onto a table prior to a behavioral tail-flicking test. After rats were habituated to the test environment for 1 h, the mechanical sensitivity of the tail was determined based on the tail withdrawal response evoked by the application of a 0.2 g (1.96 mN) von Frey hair filament. The most sensitive spot of the tail was first determined for each animal by systematically rubbing various areas of the tail with the shank of the von Frey hair; these spots were marked with a sharp marking pen. Then, each spot was challenged ten times with the von Frey hair filament at 10- to 20-s intervals. The occurrence of tail withdrawal in response to the stimulation was expressed as a percentage of trials, which served as an index of mechanical sensitivity following peripheral nerve injury. The surgery was performed on rats that were not responsive to the initial mechanical stimulation. Each rat was anesthetized with an intraperitoneal injection of Zoletil 50^® ^(50 mg/kg), after which the left superior caudal trunk was exposed, freed from the surrounding tissues and transected at the level between the S3 and S4 spinal nerves. To prevent possible rejoining of the proximal and distal ends of the severed trunk, an approximately 1-mm-long section of the trunk was removed from the proximal end. This surgery eliminated the S1-S3 spinal nerve innervation of the tail via the superior caudal trunk. Behavioral tests for signs of neuropathic pain (mechanical allodynia) were performed at 1 week after surgery. Only rats showing greater than 80% mechanical allodynia were considered to conform to the animal model for neuropathic pain.

### Isolation of single PAG neurons with synaptic boutons

The mechanical dissociation of single PAG neurons with functioning synaptic boutons was performed by using the technique described previously [38,41-43]. In brief, rats were decapitated under Zoletil 50^® ^anesthesia (50 mg/kg). The brains were removed, and transverse slices (350-μm thickness) were made with a microslicer (DTK-1000, DSK, Kyoto, Japan). Slices were preincubated in an incubation solution that had been well saturated with 95% O_2 _and 5% CO_2 _at room temperature (22-25°C) for at least 1 h before mechanical dissociation. For dissociation, slices were transferred to a 35 mm culture dish (Primaria 3801; Becton Dickinson, Rutherford, NJ, USA), and the ventrolateral region of the PAG was identified under a binocular microscope (SZ-ST, Olympus, Tokyo, Japan). Mechanical dissociation was performed using a custom-built vibration device and a fire-polished glass pipette oscillating at approximately 20-50 Hz (1-2 mm). The tip of the fire-polished glass pipette was lightly touching the surface of the ventrolateral PAG region with a micromanipulator and was vibrated horizontally for approximately 2 min. Slices were removed, and the mechanically dissociated neurons were allowed to settle and adhere to the bottom of the dish for 15 min. The isolated neurons retained short portions of their proximal dendrites.

### Electrical measurements

Electrical recordings were performed in the conventional whole-cell patch-clamp recording mode [44] under voltage-clamp conditions at holding potential (V_H_) of -60 mV. Patch pipettes were made from borosilicate capillary glass (1.5 mm outer diameter; 1 mm inner diameter; G-1.5; Narishige, Tokyo, Japan) in two stages on a vertical pipette puller (PP-83; Narishige, Tokyo, Japan). The resistance of the recording pipettes that were filled with internal solution was 5-6 MΩ. The patch pipette was positioned on the neuron using a water-driven micromanipulator (WR-60; Narishige, Tokyo, Japan). Neurons were visualized under phase contrast on an inverted microscope (IX-70, Olympus, Tokyo, Japan). Electrical stimulation, voltage control, current recording, and filtration of current (at 1 kHz) were obtained with an EPC-9 patch-clamp amplifier (EPC-9, HEKA Electronik, Lambrecht, Germany) linked to a PC controlled by HEKA software. Current and voltage were monitored continuously on a computer monitor for the EPC-9 amplifier and displayed on a paper chart linearcorder (WR3320, Graphtec, Yokohama, Japan). Membrane currents were digitized at 5 kHz with an ITC 16 board (HEKA Electronik, Lambrecht, Germany), and stored on a computer equipped with pCLAMP (version 8.0, Axon Instruments Inc., Burlingame, CA, USA). During recordings, -70 mV hyperpolarizing step pulses (30 ms in duration) were periodically delivered to monitor access resistance. All experiments were performed at room temperature (22-25°C).

### Drugs and solutions

Zoletil 50^® ^(tiletamine HCl 125 mg/5 ml + zolazepam HCl 125 mg/5 ml) was purchased from Virbac (Carros, France). Potassium phosphate monobasic, *N*-2-hydroxyethylpiperazine-*N'*-2-ethanesulfonic acid (HEPES), dimethyl sulfoxide (DMSO), ethylene glycol-bis (β-aminoethylether)-*N,N,N'N'*-tetraacetic acid (EGTA), tetraethyl ammonium chloride (TEA), BaCl_2_, CsCl, magnesium sulfate, magnesium chloride, Na-GTP, Mg-ATP, tetrodotoxin (TTX), 6-cyano-7-nitroquinoxaline-2,3-dione (CNQX), _DL_-2-amino-5-phosphonovaleric acid (_DL_-AP-5), [D-Ala^2^,N-MePhe^4^,Gly^5^-ol]enkephalin (DAMGO), naloxone HCl, (-)-bicuculline methochloride, Cs-methanesulfonate and cadmium chloride were purchased from Sigma Chemical Co. (St. Louis, MO, USA). CNQX was dissolved in DMSO at 10 mM as a stock solution. Drugs were added to the standard external solutions at the final concentrations indicated in the Result section and the vehicle concentrations never exceeded 0.01%. Drugs were superfused using a rapid application system termed the "Y-tube method" that has been described elsewhere [45,46]. The incubation solution had the following composition (in mM): 124 NaCl, 5 KCl, 1.2 KH_2_PO_4_, 1.3 MgSO_4_, 2.4 CaCl_2_, 10 glucose, and 24 NaHCO_3_. The pH was adjusted to 7.4 by continuous bubbling with 95% O_2 _and 5% CO_2_. The standard external solution had the following composition (in mM): 150 NaCl, 5 KCl, 1 MgCl_2_, 2 CaCl_2_, 10 glucose, and 10 *N*-2-hydroxyethylpiperazine-*N'*-2-ethanesulfonic acid (HEPES). The pH was adjusted to 7.4 with tris-hydroxymethylaminomethane (Tris-base). The internal pipette solution for the recording of miniature inhibitory postsynaptic current (mIPSC) had the following ionic composition (in mM): 110 CsCl, 30 TEA-Cl, 5 EGTA, 5 Mg-ATP, 0.4 Na-GTP, and 10 HEPES. The pH was adjusted to 7.2 with Tris base. To isolate spontaneous mIPSCs, external solutions routinely contained 300 nM TTX, 1 μM CNQX, and 10 μM AP-5 to block voltage-dependent Na^+ ^channels and glutamatergic excitatory synaptic currents.

### Statistical analysis

Spontaneous mIPSCs were analyzed using the MiniAnalysis program (Synaptosoft Inc., Leonia, NJ, USA). Kaleida Graph software (Synergy Software, Reading, PA, USA) was used for curve fitting. Spontaneous events were initially detected automatically using an amplitude threshold of 5 pA (for mIPSC) and then visually accepted or rejected on the basis of their rise and decay times. Events with brief rise times (0.5-1.5 ms) and decay times that were fitted by a single-exponential function were selected for fast current detection. Averaged current frequency and amplitude were normalized to the control conditions and were provided as means ± S.E.M. Differences in current amplitude and frequency between each single neuron were tested with Student's paired two-tailed *t-*test using absolute values. Fisher's Exact test was performed to see if there was a contingency between the two kinds of classification. Difference in amplitude distributions of miniature currents obtained from a single neuron were examined by constructing all-point cumulative probability distributions and compared using the Kolmogorov-Smirnov (K-S) test. Values of *P *< 0.05 were considered significant. The mIPSC kinetics were fitted by two exponential functions for further detailed analysis and were described as their decay phases with time constants and area under the current. The weighted mean decay time constant (τ_m_) was calculated as τ_m _= (A_fast_×τ_fast _+ A_slow_×τ_slow_)/(A_fast_+A_slow_), where τ_fast _and τ_slow _are the respective time constants, and A_fast _and A_slow _are the current amplitude constants. Each parameter was compared using Student's paired two-tailed *t-*test. Values of *P *< 0.05 were considered significant.

## Authors' contributions

ETH, YK, and YWC designed the experiments. ETH, YK, and JJL made the neuropathic pain animal model, performed the experiments and analyzed the data. ETH and YWC wrote the manuscript. All authors read and approved the final manuscript.
